# Complete Genome Analysis of Flavobacterium psychrophilum Strain FPRT1, Isolated from Diseased Rainbow Trout (Oncorhynchus mykiss) in South Korea

**DOI:** 10.1128/MRA.00151-21

**Published:** 2021-03-25

**Authors:** Jiyeon Park, HyeongJin Roh, Yoonhang Lee, Junewoo Park, Do-Hyung Kim

**Affiliations:** a Department of Aquatic Life Medicine, Pukyong National University, Busan, Republic of Korea; Georgia Institute of Technology

## Abstract

Here, we report the complete genome sequence of Flavobacterium psychrophilum FPRT1, isolated from the spleen and kidney of diseased rainbow trout (Oncorhynchus mykiss). Whole-genome sequencing was performed using the PacBio RS II platform, which yielded a circular chromosome of 2,795,347 bp harboring 2,895 protein-coding genes.

## ANNOUNCEMENT

Flavobacterium psychrophilum is a Gram-negative bacterial pathogen that causes bacterial cold-water disease and rainbow trout fry syndrome (1, 2). The clinical signs of diseased rainbow trout in this study included ulcerative lesions in the mandible, pale gills (anemia), exophthalmia, and increased mucus secretion. Fifty rainbow trout (body weight, ∼70 g) were purchased from a fish farm in South Korea. Fish were acclimated in a 500-liter tank and maintained at 15°C. Freshly dead fish were aseptically dissected, and swabbed materials derived from the kidney and spleen were placed onto tryptone-glucose-yeast extract (TGYE) agar. Bacterial cultures isolated as a pure culture were used to extract DNA using the AccuPrep genomic DNA extraction kit (Bioneer, South Korea). PCR was performed using the primers 27F (5′-AGAGTTTGATCMTGGCTCAG-3′) and 1492R (5′-TACGGYTACCTTGTTACGACTT-3′) for 16S rRNA gene sequencing. The organism was identified as F. psychrophilum (identity, 100%). F. psychrophilum FPRT1 growth required at least 72 h of incubation on TGYE agar at 15°C under aerobic conditions and was stored in TGYE broth supplemented with 20% glycerol at −80°C. Antibiotic susceptibility testing according to the guidelines of the Clinical and Laboratory Standards Institute (3) showed inhibition zones were not formed for oxolinic acid, trimethoprim, and sulfamethoxazole, indicating that this strain was resistant to sulfa drugs and the quinolone.

A single colony of F. psychrophilum FPRT1 was inoculated into TGYE broth and incubated at 15°C for 36 h for DNA extraction. Genomic DNA of FPRT1 was extracted using a Wizard genomic DNA purification kit (Promega, USA) according to the manufacturer’s protocol. The whole-genome sequencing was performed using the PacBio RS II (Pacific Biosciences, USA) platform. Library preparation was performed using single-molecule real-time (SMRT) cell 8Pac V3 and DNA polymerase binding kit P6 (Pacific Biosciences). Genomic DNA was sheared with g-TUBE (Covaris, USA) into a 20-kb size and purified using AMPure PB magnetic beads (Beckman Coulter, USA). A bioanalyzer 2100 instrument (Agilent, USA) was used to determine the actual size distribution. After subread filtering of the PacBio raw data (minimum polymerase read quality, 0.80), 132,029 PacBio subreads (average subread length, 10,730 bp; subread *N*_50_, 15,690 bp) of FPRT1 were generated. Raw sequences were assembled *de novo* using Hierarchical Genome Assembly Process 3 (HGAP3), and error correction was conducted using Quiver (4). When both ends of the contig overlapped, contigs were connected to form a circular shape, and overlapped regions were manually trimmed. The coding DNA sequence (CDS), tRNA, and rRNA genes on each contig were predicted and annotated using Rapid Annotations using Subsystems Technology (RAST) and SEED viewer (5, 6). Antibiotic resistance-related genes were searched in the Comprehensive Antibiotic Resistance Database (CARD) (7). Default settings were used for all software.

**FIG 1:**
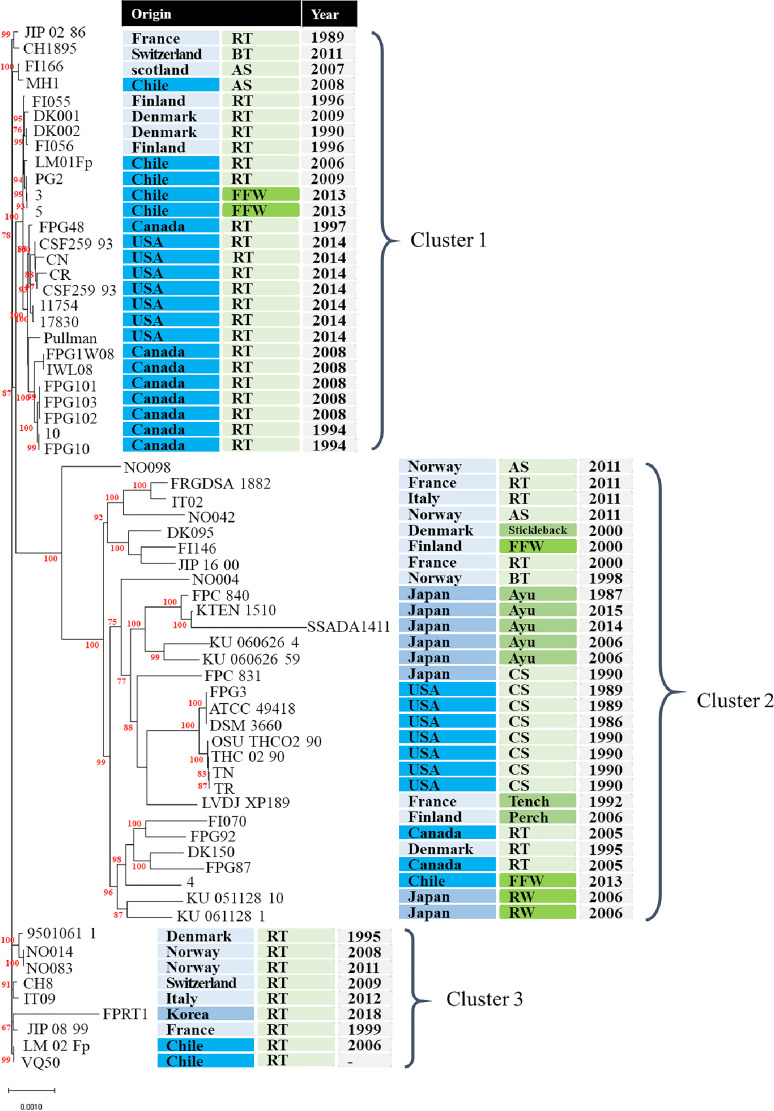
Neighbor-joining phylogenetic tree of 65 strains of Flavobacterium psychrophilum based on 831 orthologous genes. Bootstrap values (only >50%) from 500 replicates are indicated at each node. Abbreviations: RT, rainbow trout; BT, brown trout; AS, Atlantic salmon; CS, Coho salmon; FFW, freshwater fish farm water; RW, river water.

The genome consisted of 2,795,347 bp in a single circular chromosome with an overall G+C content of 32.6%, containing 2,895 CDS, 49 tRNA, and 14 rRNA genes. Additionally, phylogenetic analysis (8) based on 831 orthologous genes retrieved from 65 publicly available F. psychrophilum genome sequences showed that they were phylogenetically divided into 3 clusters (Fig. 1). Our strain was most closely related to isolates originating from some European countries and Chile within cluster 3. In addition, strain FPRT1 harbors several antibiotic resistance-related genes, such as *emrA* and *emrB*, that encode multidrug efflux pumps in the MFS family (9). This is the first report of genomic sequencing of F. psychrophilum from infected fish in South Korea. In the era of whole-genome sequencing, this study might provide important epidemiological information on F. psychrophilum.

### Data availability.

The whole-genome sequence of F. psychrophilum FPRT1 has been deposited in the GenBank database with the accession number CP059061.1. The raw sequencing data are available under accession number SRR11814644. The BioProject and BioSample numbers are PRJNA633452 and SAMN14943929, respectively.
